# Electronic structure of Schiff-base peroxo{2,2′-[1,2-phenyl­enebis(nitrilo­methanylyl­idene)]bis­(6-meth­oxy­phenolato)}titanium(IV) monohydrate: a possible model structure of the reaction center for the theoretical study of hemoglobin

**DOI:** 10.1107/S205225252100004X

**Published:** 2021-02-18

**Authors:** Júlia Adamko Kožíšková, Martin Breza, Marián Valko, Peter Herich, Lukáš Bučinský, Jozef Kožíšek

**Affiliations:** a Institute of Physical Chemistry and Chemical Physics, Slovak University of Technology in Bratislava, Radlinského 9, Bratislava SK-81237, Slovakia

**Keywords:** peroxo bond activation, homolytic and heterolytic O—O bond weakening, electronic structure, charge density, quantum crystallography, DAFH, hemoglobin, crystal engineering, charge spin and momentum densities, molecular crystals, structure prediction

## Abstract

An extensive characterization of [Ti(C_22_H_18_N_2_O_6_)]·H_2_O was performed by topological analysis according to Bader’s quantum theory of atoms in molecules (QTAIM) from the experimentally (multipole model) and theoretically (DFT) determined electron density. To the best of our knowledge, this study is the first example of an experimental electronic structure of a coordination compound in which a peroxo anion is bonded to a 3*d* central atom. The titanium coordination polyhedron could be described as a deformed tetrahedral pyramid if the midpoint of the peroxide O—O bond (*side-on* mode) is considered to be in the quasi-apical position. According to the multipole model (*MM*) results, the titanium atom has a positive QTAIM charge of 2.05 e^−^ which does not correspond to the formal Ti (IV) oxidation state. On the other hand, the peroxo oxygen atoms O(1) and O(2) have *MM* QTAIM charges of −0.27 and −0.12, respectively. This asymmetric charge density distribution on the peroxo oxygens is in agreement with the distorted orientation of the O_2_ moiety with respect to the titanium atom. Despite the fact that the overall *MM* charge of the O_2_ moiety is more remote from the formal −2 charge than from neutral O_2_, the O—O distance remains close to that in the peroxo O_2_^2−^ anion. In the case of DFT results, the titanium atom charge is also found to be close to +2, the O_2_^*x*−^ moiety charge is around −1, the optimized O—O distance is shorter by only *ca* 0.04 Å than the experimental value of 1.5005 (16) Å, and the DFT *d*-populations on titanium are found to be lower than the experimental *MM* value. This study is the first experimental electronic structure of a transition metal peroxo complex.

## Abstract 2

The experimental electron-density distribution in a titanium peroxo complex has been studied and compared with theoretical results.

## 1. Introduction   

All living organisms requiring molecular oxygen for life mediate four-electron reduction of oxygen to water (Valko *et al.*, 2004 ▸). In the course of the reduction process, the energy formed is utilized by aerobic organisms maintaining life on the Earth. Molecular oxygen is in triplet ground state with two parallel unpaired electrons (*S* = 1) which represents the most stable oxygen form. The first step of the reduction cascade, representing reduction of molecular oxygen to superoxide radical anion is a rather unfavorable, endergonic reaction (∼33000 J mol^−1^) (Valko *et al.*, 2005 ▸). The molecular oxygen biradical has two parallel electrons in antibonding π orbitals and therefore its reactions with organic molecules, in which all electrons are paired and in closed-shell systems, are spin forbidden. To overcome thermodynamic and electronic restrictions in the process of reduction of molecular oxygen, nature has evolved a variety of metallo-enzymes to store and transport molecular oxygen as well as catalyze its conversion to more reduced forms.

In their structure metallo-enzymes contain integrally anchored transition metal ions with unpaired *d* electrons (Rebilly *et al.*, 2015 ▸). Paramagnetic metal ions in various electronic states can activate molecular oxygen in its ground state and make the process of reduction more feasible. Interaction of molecular oxygen with metal centers of metalloproteins or coordination compounds containing transition metals changes the thermodynamics as well as the kinetics of oxygen reduction. Upon interaction of metal ions with molecular oxygen a variety of intermediates such as superoxo, hydro­peroxo and oxo-species are formed (Valko *et al.*, 1995 ▸). These metal–oxygen adducts are usually less reactive and more stable than typical organic radicals such as reactive oxygen species (ROS), or reactive nitro­gen species (RNS).

In the past two to three decades, titanium has been increasingly used in materials that improve the life quality of humans. Titanium is a key component of prosthetics and therefore this element is in direct contact with biological fluids and/or tissues. Thus, the interaction of titanium with physiological target molecules of various molecular weights and biological functions may occur extensively. This raises the question of what types of interaction may develop between the metal ion (in various oxidation states) and surrounding biological tissues. Such interactions may play an important role in the activation of signal-transduction pathways, activation of enzymes and expression of genes which in turn may explain the clinical symptoms described in clinical investigations (Dakanali *et al.*, 2003 ▸).

Interaction of titanium complexes with di­oxy­gen is of importance for better understanding the nature of metal–oxygen interactions, the reversible reaction of carbon dioxide with metal oxides, development of more effective titanium di­oxy­gen species with improved photocatalytic activity and other properties (De Lile *et al.*, 2017 ▸).
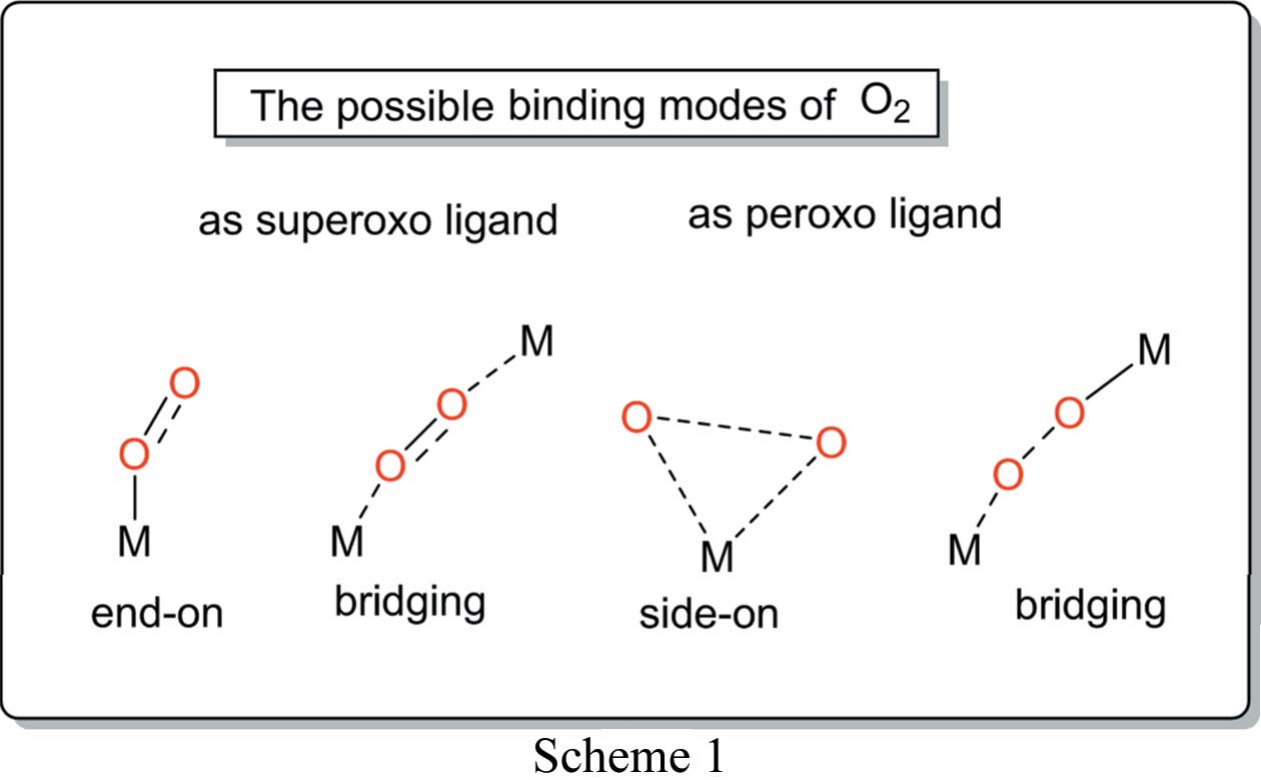


The interaction of di­oxy­gen with titanium is a result of the interaction between the oxygen antibonding π^*^ orbital and the non-bonding *d* orbitals of the metal ion. Generally, there are four possible modes of di­oxy­gen binding to a metal center: *end-on*, *side-on* and *bridge* (Scheme 1). Titanium capable of binding peroxides can activate them toward oxidation of a variety of substrates and consequently decompose them (DiPasquale *et al.*, 2002 ▸). Interest related to Ti(IV)-peroxo species has been associated with reactive superoxide radical anion traps and various reactive oxygen species formed during inflammatory processes (Tengvall *et al.*, 1991 ▸).

In the Cambridge Structural database the crystal structures of transition metals (*M*) with one, two or three peroxo groups (*side-on* mode, different geometries) may be found as follows:

(i) one peroxo group for *M* = V (80 structures), Mo (40 structures), Ti (33 structures), Ru (25 structures), Rh, W, Ir (19 structures), Nb (6 structures), and Os, Mn, Co, Ni, Ta, Hf and Re (one structure);

(ii) two peroxo groups for *M* = V, Nb, Mo, W, Re, Hf and Ir;

(iii) three peroxo groups for *M* = V, Nb and Ta.

From 33 Ti crystal structures with one coordinated peroxo group, we will briefly point out those that are *side-on* coordinated for comparison purposes with the title compound. The crystal structure of [Sr(H_2_O)_7_][Ti(O_2_)(edta)]·H_2_O (KOGHUK, Deng & Zhou, 2008 ▸) has the peroxo-group distances Ti—O(1), Ti—O(2) and O(1)—O(2) of 1.833 (2), 1.844 (2) and 1.453 (3) Å, respectively. In crystal structures of titanium ethyl­enedi­amine with chromophore [Ti(O_2_)O_3_N_2_] [VINGAB, VINGEF, VINGIJ and VINGUV (Zhou *et al.*, 2007 ▸)] the analogous Ti–O(ethyl­enedi­amine) distances are in the interval 1.948–2.086 Å and those of Ti(1)–N(ethyl­enedi­amine) are in the interval 2.192–2.300 Å.

Through the experimental electron density, chemical bonding can be understood from experimental and theoretical points of view. More than 99.9% of single-crystal structure determinations are based on the spherical atom model. These studies are able to determine the bond distances and angles between atoms in the molecule, as well as all interatomic interactions [of course with small systematic errors due to the independent atom model (IAM)]. On the other hand, using the Hansen–Coppens multipole formalism (Hansen & Coppens, 1978 ▸) for accurate diffraction data with satisfactory resolution, providing all necessary corrections, it is possible to obtain a valence electron distribution that reflects bonding properties and interactions in the studied molecule. Despite the low suitability factor (Coppens, 1997 ▸), recently published papers of experimental electronic structures of 3*d*-coordination compounds show reasonable results (Schmøkel *et al.*, 2013 ▸; Herich *et al.*, 2018*a* ▸,*b* ▸; Fukin *et al.*, 2019 ▸; Gao *et al.*, 2019 ▸; Scatena *et al.*, 2019 ▸).

Targeted preparation of compounds with desirable properties requires a deep understanding of the relationship between the structure and properties of the compounds studied, which are closely related to their chemical bond. The goal of our study is to elucidate weakening of the O—O bond in the studied peroxo complex which may facilitate a subsequent redox reaction of the coordinated peroxo group.

## 2. Experimental   

### 2.1. Material and methods   

#### 2.1.1. Synthesis and crystal growth   

All chemicals were purchased commercially and were used as received without further purification. The synthesis was carried out in three steps. In the first step, we prepared the Schiff base from *o*-vanillin (0.304 g, 2 mmol, *o*-vanillin 99%, Alfa Aesar) and *o*-phenyl­endi­amine (0.108 g, 1 mmol, *o*-phenyl­enedi­amine 99%, Sigma–Aldrich) in 50 ml methanol [methanol (p.a.) was a product of CENTRALCHEM]. The Schiff base solution was stirred for 30 min. In the second step, titanium (IV) butoxide [0.340 g, 1 mmol (in a 5% surplus), titanium (IV) butoxide 97%, reagent grade, Sigma–Aldrich] was added to the Schiff base solution with vigorous stirring. The yellow Schiff base solution turned dark orange. The precipitated TiO_2_ was filtered from the solution. Finally, we added H_2_O_2_ [0.034 g, 1 mmol (0.11 g, 30% solution), hydrogen peroxide 30%, reagent grade ISO, Sigma–Aldrich] and the dark orange solution turned light orange. The orange crystals dropped out of solution after 1 d in the refrigerator. After crystallization a single crystal suitable for X-ray was selected.

#### 2.1.2. Data collection   

A high-quality yellow rod-shaped single crystal with the dimensions 0.150 × 0.050 × 0.045 mm was measured on a Eulerian four-circle diffractometer Stoe STADIVARI with a Dectris Pilatus 300 K detector, Incoatec IμS Ag microfocus source (Ag *K*α, λ = 0.56083 Å) at 100 K using a nitro­gen gas open-flow Cobra cooling system from Oxford Cryosystems. Two detector positions for 64 omega scans (2θ = 4.5 and 89.3°) with a 0.5° frame width were used. The exposure time was 200 s. The maximum resolution reached at this experimental setting was *d* = 0.399 Å and sin(θ)/λ = 1.253 Å^−1^. The data reduction was performed using *X-Area* Integrate (version 1.73.1) and *X-Area X-Red32* (version 1.65.0.0; Stoe & Cie, 2018 ▸). For absorption correction a crystal-shape model with eight faces was employed. The average redundancy was 9.9, *R*_int_ and *R*_σ_ were 0.0696 and 0.0307, respectively. From the data reduction we obtained direction cosines and TBAR (distance of the primary and diffracted beam through the crystal) first as described previously (Kožíšek *et al.*, 2002 ▸; Herich *et al.*, 2018*a* ▸). Details of the X-ray diffraction experiment conditions and the crystallographic data are given in Table 1 ▸. As the symmetry-equivalent data were collected with a different value of TBAR, all non-averaged data were used in the refinements.

### 2.2. Electron density refinements   

The structure was solved by the dual-space algorithm implemented in *SHELXT* (Sheldrick, 2015*a* ▸). The IAM was refined using *SHELXL* (Sheldrick, 2015*b* ▸) and the graphical user interface *Olex2* (Dolomanov *et al.*, 2009 ▸). For *MM* refinement the Hansen–Coppens model (Hansen & Coppens, 1978 ▸) was used. The total atomic density ρ(**r**) in this approach is divided into three contributions:
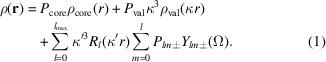
The first two components describe the spherical *core* and spherical *valence* electron density (Hansen & Coppens, 1978 ▸) and the third term describes the aspherical deformation of the valence electron density. *R*_*l*_ are normalized Slater-type radial functions and *Y*_*lm*__±_ are the density normalized real spherical harmonics. The parameters κ and κ′ are responsible for contraction/expansion of the spherical and aspherical valence parts.

*MM* refinement calculations were based on *F* ^2^ refinements using the *XD2016* (Volkov *et al.*, 2016 ▸) suite of programs and the low-temperature (100 K) X-ray diffraction data. The least-squares procedure accounted only for reflections with *I* > 3σ(*I*) using the Su-Coppens (Su & Coppens, 1998 ▸) (SCM) wavefunctions databank. Details on the refinements are provided in S1 of the supporting information. An error analysis revealed that there is quite a large fluctuation of the scale factors *versus* sin(θ)/λ. The residual density calculated by fast Fourier synthesis (XDFFT) for all diffractions is 3.43 e Å^−3^ at 0.03 Å from the titanium atom and −1.21 e Å^−3^ at 0.48 Å from the titanium atom with a mean value of 0.155 e Å^−3^. We introduced 19 scale factors into the multipole refinement, one for each group, as suggested by Niepötter *et al.* (2015 ▸). After the complete procedure of multipole refinement the residual density decreased to 2.38 e Å^−3^ at 0.02 Å from the titanium atom and −0.82 e Å^−3^ at 0.45 Å from the titanium atom with a mean value of 0.134 e Å^−3^. As integration in the atomic basin gives much lower charges for peroxo-oxygen atoms, different scattering curves (neutral for O_2_^2−^ and O^−^ for other oxygen atoms) were introduced. Multipole refinement was repeated with slightly lower *R* values obtained (3.8622% versus 3.8544%). Small changes were observed for charges of peroxo-oxygen atoms, they are more negative and other oxygen atoms less negative (see Table S4). Also atomic volumes for non-peroxo oxygen atoms are larger. At the end of the multipolar refinement we found that it is not necessary to refine the secondary extinction.

The fractal plot of the residual density (Meindl & Henn, 2008 ▸) has a symmetrical shape for the entire sin*θ/λ* range of the data set with ρ_min_ = −0.77 e Å^−3^ and ρ_max_ = 0.207 e Å^−3^ (see Fig. S1 of the supporting information).

The normal probability distribution plot (Abrahams & Keve, 1971 ▸; Farrugia, 2012 ▸) shows a fairly good agreement with the assumed shape (Fig. S2 of the supporting information). The slope is 45°, the function goes through the origin and is linear in the interval from −3 to 3 (Abrahams & Keve, 1971 ▸). The variation of the scale factor with respect to the resolution is about 8% higher for the last group (see Fig. S3 (Farrugia, 2012 ▸) in the supporting information). It could be said that the error analysis has affirmed a good agreement between the experimental and calculated structure factors.

### 2.3. Quantum chemical calculations   

Geometry optimization of the neutral complex under study in singlet ground state starting from the X-ray structure and of the O_2_^*q*^ molecule, with charges *q* = 0, −1 and −2 in various spin states, was performed by employing the B3LYP hybrid functional (Becke, 1988 ▸; Lee *et al.*, 1988 ▸; Vosko *et al.*, 1980 ▸; Stephens *et al.*, 1994 ▸) with Grimme’s D3 dispersion corrections (Grimme *et al.*, 2010 ▸) and 6–311+G* basis sets from the Gaussian library (Frisch *et al.*, 2016 ▸) for all atoms. Alternatively, the same O_2_^*q*^ molecule was optimized at coupled clusters with single and double excitation (CCSD) levels of theory (Scuseria & Schaefer, 1989 ▸) with the same basis sets. The stability of the optimized structures was tested by vibrational analysis (no imaginary vibrations). The *Gaussian16* program suite (Frisch *et al.*, 2016 ▸) was used for all quantum chemical calculations. The electronic structure analysis in terms of quantum theory of atoms in molecules (QTAIM) (Bader, 1994 ▸) was performed in the *AIMAll* package (Keith, 2016 ▸) using the wavefunctions from the *Gaussian16* wfn and/or fchk files. The *d*-electron populations at titanium were obtained using natural population analysis (Carpenter & Weinhold, 1988 ▸) as implemented in *Gaussian16* (Frisch *et al.*, 2016 ▸).

The domain averaged Fermi holes (DAFH) analysis (Ponec, 1998 ▸, 2010 ▸; Ponec & Cooper, 2007 ▸; Baranov *et al.*, 2012 ▸) was performed with the *D-Grid* package (version 5.1 Kohout, 2019 ▸) to analyze the bonding interactions between the central titanium atom and the O_2_ moiety. DAFH eigenvectors have been visualized with the *IQmol* package (Gilbert, 2020 ▸).

### 2.4. (QT)AIM analysis   

The total electron densities obtained from the multipole refinement and alternatively from theoretical calculations have been analyzed within the framework of the (QT)AIM (Bader, 1994 ▸). The results were evaluated in terms of atomic charges obtained using the electron density integrated over atomic basins and bond characteristics in terms of electron density ρ at bond critical points (BCPs) corresponding to saddle points at bond paths between individual atoms, its Laplacian ∇^2^ρ can be expressed by 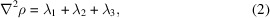
and bond ellipticity ɛ
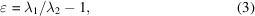
where λ_1_ < λ_2_ < 0 < λ_3_ are the eigenvalues of the electron density Hessian at BCPs. Ring critical points are saddle points with λ_1_ *<* 0 < λ_2_ < λ_3_ and cage critical points are local minima (0 < λ_1_ < λ_2_ < λ_3_) of electron density.

The BCP electron density (ρ_BCP_) is proportional to the bond strength; the value and sign of its Laplacian (∇^2^ρ_BCP_) describes the relative electron density contribution of the bonded atoms to the bond (covalent versus dative bonding); its bond ellipticity (ρ_BCP_) describes its deviation from cylindrical symmetry (such as in ideal single or triple bonds) due to its double-bond character, mechanical strain and/or other perturbations.

### 2.5. DAFH analysis   

In the case of the DAFH analysis, one can distinguish electron pairs (eigenvectors) which are broken or retained in a chosen part (domain) of a studied system (Ponec, 1998 ▸; Ponec & Cooper, 2007 ▸; Ponec *et al.*, 2010 ▸; Baranov *et al.*, 2012 ▸). In our case, we choose the O_2_ moiety as the domain (using QTAIM atomic basins) to quantify the strength of dative interactions between Ti and O_2_. Each DAFH eigenvector has an assigned eigenvalue (occupation) in the range 0–2, which represents the amount of electron density inside the chosen domain. Eigenvalues close to 2 correspond to a retained non-bonding electron pair within the domain, whereas eigenvalues below 2 represent that some part of the electron density is outside the domain due to bonding or dative interactions (and possibly also antibonding ones). For instance, an eigenvalue of 1.6 suggests a dative interaction, with 1.6 out of 2 electrons of a given electron pair (DAFH eigenvector, Fermi hole) being the part of the chosen domain. Eigenvectors with eigenvalues below 0.2 or 0.1 are mostly excluded from consideration, representing rather a numerical noise of the method. In addition, DAFH analysis uses an isopycnic localization (Cioslowski, 1990 ▸) to provide more useful information with respect to chemical intuition.

## 3. Results and discussion   

### 3.1. Structure description   

**Figure 1:**
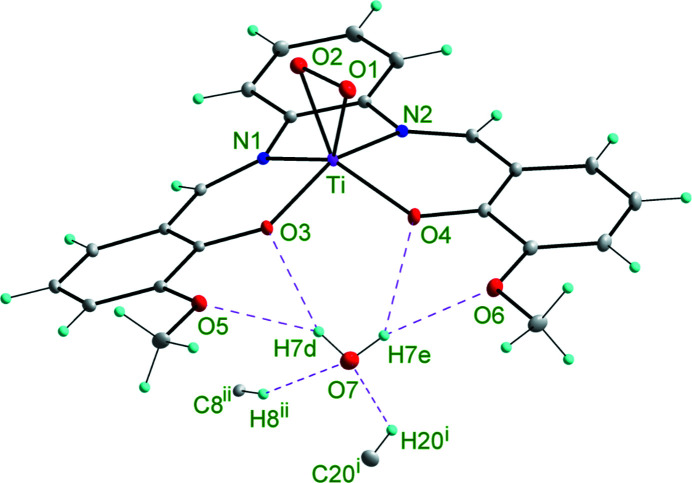
*ORTEP* plot of the title compound. Thermal ellipsoids are drawn at 30% probability. Symmetry codes used: (i) *x*, −1 + *y*, *z*; (ii) 1 − *x*, 1 − *y*, 1 − *z*.

The coordination polyhedron of the central titanium atom, described by chromophore [Ti(O_2_)O_2_N_2_], is a deformed tetragonal pyramid (Fig. 1 ▸) with the peroxo anion in the apical position (*side-on* mode) and two oxygen and two nitro­gen atoms in the basal plane. The titanium atom is shifted from the plane defined by basal plane atoms O(1), O(2), N(1) and N(2) by 0.671 Å towards the peroxo anion. The angle between the basal plane and the plane defined by atoms O(1), O(2) and Ti is 86.45°. There are no similar crystal structures in the Cambridge Structural Database (Groom *et al.*, 2016 ▸) except one (Guilard *et al.*, 1978 ▸) with the chromophore [Ti(O_2_)N_4_] (*side-on* bonding mode). The titanium central atom is above the basal plane by 0.621 Å and the angle between the corresponding planes is 89.09°, defined above (Guilard *et al.*, 1978 ▸). The interatomic distances in this compound are 1.822 (4), 1.827 (4) and 1.445 (5) Å, and in the title compound are 1.8699 (12), 1.8813 (11) and 1.5018 (16) Å for the Ti—O(1), Ti—O(2) and O(1)—O(2) bonds, respectively. It should be mentioned that the IAM model *SHELXL* refinement of the title compound gives the above values of 1.8522 (7), 1.8685 (6) and 1.4674 (9) Å, respectively. The O(1)—O(2) bond distances from the IAM model for both crystal structures points to an inadequacy of the model when comparing against the *MM* derived distance. On the other hand, the O(1)—O(2) bond distance from the *MM* refinement corresponds well with the value 1.499 (2) Å found in the solid sodium peroxide hydrate in which each oxygen atom is surrounded by four hydrogen bonds (Hill *et al.*, 1997 ▸). The title crystal structure is stabilized by four intramolecular and two intermolecular hydrogen bonds (Table S1). It is important to state that interatomic distances from AIM refinement could suffer from systematic errors and the *MM* refinement obtains more accurate values. The interatomic distances and angles are shown in Table S2.

The *MM* refinement achieved a significant improvement of the agreement between the experimental and calculated structure factors when compared with ordinary IAM structure refinement. Furthermore, the accuracy in the interatomic distances is increased by an order of magnitude compared with a routine IAM *SHELXL* refinement.

### 3.2. Topology of *MM* and DFT charge densities   

The aim here was to characterize the studied crystal structure and topological properties of the *MM*-refined experimental charge density and to make a comparison with density functional theory (DFT). A further task was to detect the amount of electron density transfer from the peroxide anion to the rest of the molecule and thus characterize the changes of the peroxo O—O bond due to the coordination to Ti(IV).

**Figure 2:**
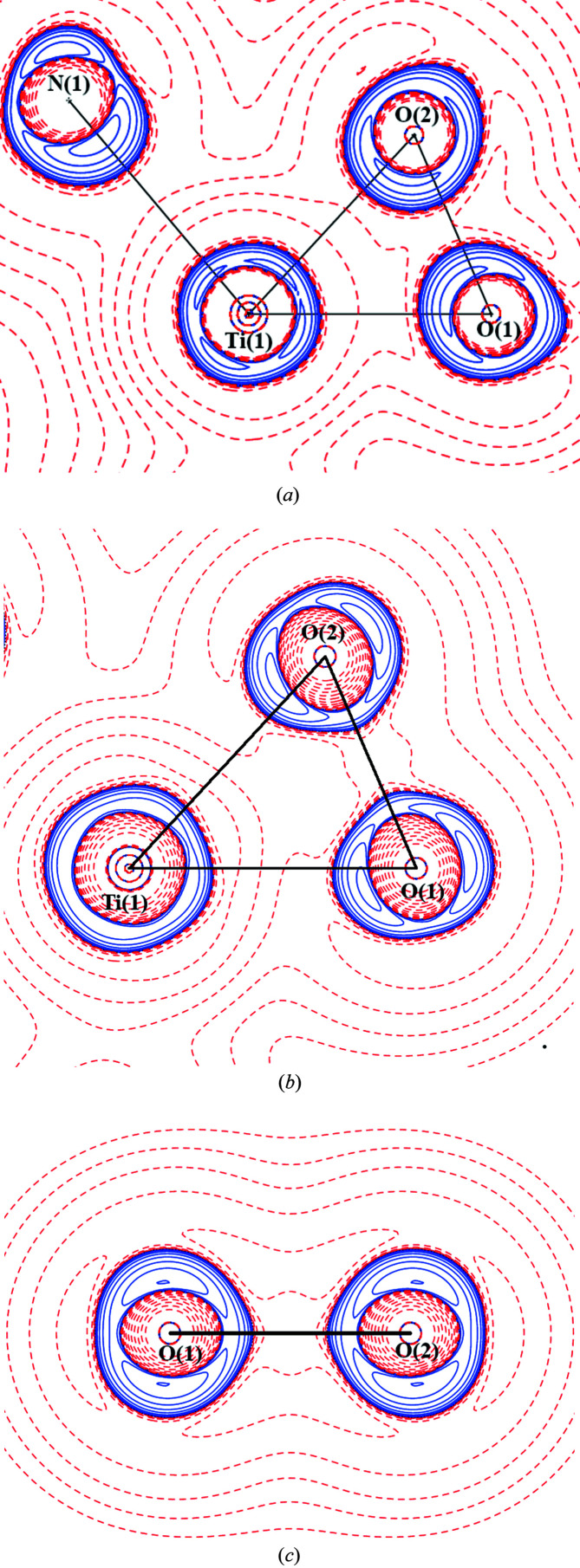
(*a*) Experimental Laplacian distribution *L*(*r*) ≃ ∇^2^ρ(*r*) in the Ti(1)—O(1)—O(2) plane; (*b*) theoretical Laplacian distribution *L*(*r*) ≃ ∇^2^ρ(*r*) in the Ti(1)—O(1)—O(2) plane; (*c*) theoretical Laplacian of the free O_2_^2−^ anion. Contours are drawn at −1.0 × 10^−3^, ±2.0 × 10^*n*^, ±4.0 × 10^*n*^, ±8.0 × 10^*n*^ (*n* = −3, −2 −1, 0, +1, +2 +3) e Å^−5^, with positive contours drawn with a solid blue line and negative contours with a dashed red line.

According to the QTAIM BCP descriptors obtained for the *MM*-refined charge density, the strongest coordination bonds are Ti—O(1) and Ti—O(2) where ρ_BCP_ and ∇^2^ρ_BCP_ have the highest values (Table 2 ▸). Ti—O(3) and Ti—O(4) bonds from phenyl­enedi­amine are weaker and the coordination bonds Ti—N(1) and Ti—N(2) from vanillin are the weakest. This is also confirmed in the theoretical results (see Tables 2 ▸, S2 and S3). All experimental and theoretical values of Ti—O and Ti—N coordination bonds are in good agreement with each other except the ellipticity of the Ti—O(1) and Ti—O(2) bonds in the peroxo anion. The experimental ellipticity of Ti—O(1) and Ti—O(2) bonds are about ten times higher than the theoretical ones. Such high values of ellipticity are even larger than the experimental values for cyclo­propane which are in the interval 0.61–0.67 (Bacsa & Briones, 2013 ▸). Ellipticity expresses the amount of π bonding contributions as well as the mechanical strain at a particular bond in cyclic structures. The *MM* BCP Laplacian values for the coordination bonds are found to be larger than the theoretical values, especially for the oxygen atoms, in particular O(1) and O(2), see Table 2 ▸. A similar statement is true for the O(1)—O(2) BCP Laplacian as well as the BCP electron density, see Table 2 ▸. Interestingly, the O(1)—O(2) BCP Laplacian is found to be positive, which is not consistent with a typical covalent bond. The explanation could be based on the shift of the electron density in the region between the two oxygen atoms to the titanium atom, and thus the less negative region on the oxygen nucleus interaction with the negative region which resembles the closed-shell interaction. The difference between the theoretical Laplacian of the free O_2_^2−^ anion [Fig. 2 ▸(*c*)] and the *MM* Laplacian of the coordinated O_2_^2−^ anion is that the first is symmetrical according to the center of the O—O bond, and in the case of the coordinated one, the valence shell charge concentration (VSCC) is asymmetric and shifted to the central titanium atom [Figs. 2 ▸(*a*) and 2(*b*)].

In our last paper (Vénosová *et al.*, 2020 ▸) we tested the improvement of *MM* flexibility by improving the radial functions of sulfur, oxygen and nitro­gen atoms according to the work by Dominiak & Coppens (2006 ▸). Zeta values were taken from the *JANA2006* database (Petříček *et al.*, 2014 ▸). For the C_22_H_18_N_2_O_6_Ti·H_2_O complex under study, we considered the radial function flexibility of the oxygens, but no changes were observed.

In the case of QTAIM charges, the transfer of charge density from the O(1)—O(2) moiety is found to amount to almost two electrons in the *MM*-refined results (1.61 e), whereas in the theoretical results we found a charge transfer of only one electron (0.98 e) from the O(1)—O(2) moiety, see Table S4. Still the *MM* and DFT QTAIM charges of Ti are both close to two. Further differences in the *MM* and DFT charges are found also for the remaining oxygen atoms, with the *MM* charges being more negative. The more negative the *MM* charges of O(5) and O(7) atoms counterbalance the larger charge transfer from the O(1)—O(2) moiety, when comparing to DFT QTAIM charges. Experimental results take into account intermolecular hydrogen bonds and non-covalent interactions (Table S5) from adjacent molecules which could stabilize a larger charge transfer from the O(1)—O(2) moiety, whereas DFT results (see below) reflect the electronic structure of an isolated molecule.

**Figure 3:**
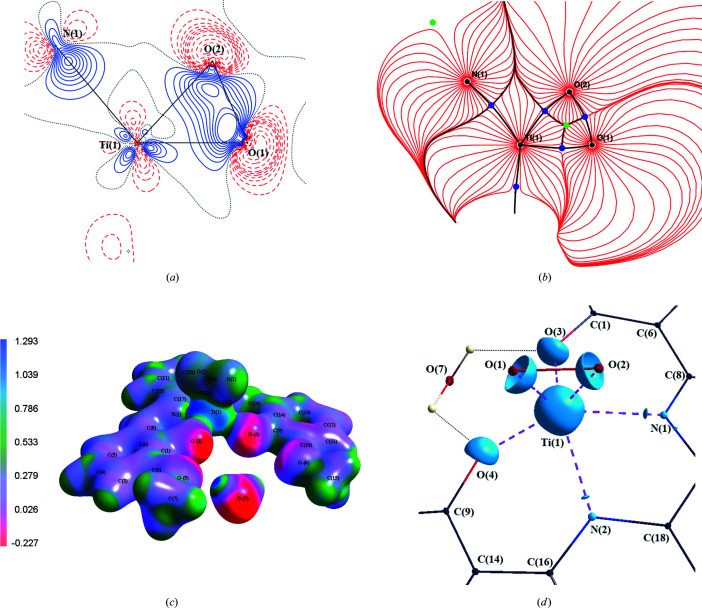
(*a*) Static electron deformation density in the plane defined by the atoms Ti(1)—O(1)—O(2). Contour spacing 0.1 e Å^−3^, with positive contours drawn with a solid blue line and negative contours with a dashed red line; (*b*) gradient field trajectory plot of electrostatic potential in the plane Ti—O(1)—O(2); (*c*) experimental electrostatic potential on the 3D-isosurface of the experimental electron density (0.3 e Å^−3^); (*d*) three-dimensional plot (Hübschle & Dittrich, 2011 ▸) of the Laplacian of electron density around Ti at an isosurface value of 80 e Å^−5^.

Furthermore, we will consider the *MM* deformation density [Fig. 3 ▸(*a*)] and Laplacian for the Ti—O(1)—O(2) plane for comparison with the QTAIM results [Fig. 2 ▸(*a*) and 2(*b*)]. A classical coordination bond is understood as a bond where the lone electron pair of a single donor atom and the depopulated *d*-orbital of the central atom are involved. In the studied complex, one finds a perpendicular coordination of the O_2_ moiety with respect to Ti. Hence, *MM* deformation densities in the Ti—O(1)—O(2) plane uncover a superposition of a σ-like interaction in the O_2_ moiety, with the coordination bond contributions between the depopulated *d*(Ti) orbitals and the fully occupied π(O_2_) and π*(O_2_) orbitals, see Fig. 3 ▸(*a*). Thus, the deformation O(1)—O(2) bonding density appears to be shifted towards Ti. The Laplacian for the Ti—O(1)—O(2) plane shows also that the O(1) and O(2) maxima are tilted off the actual O(1)—O(2) axis and build a VSCC towards titanium on each oxygen atom. This Laplacian map also confirms the positive Laplacian region between the O(1)—O(2) atoms (Table 2 ▸).

Experimentally found VSCCs consistently found in the figures of electrostatic potential (BCP) are placed outside the triangle Ti(1)—O(1)—O(2), the gradient field trajectory plot of electrostatic potential, the static deformation map and the map of Laplacian [Fig. 3 ▸(*b*)].

Last but not least, Table S6 presents the *MM* and DFT *d*-orbital populations. Generally, the individual *MM* *d*-orbital populations are higher by approximately 0.3 e^−^ than those of DFT (except *d*_*x*^2^__−__*y*^2^_ and *d_xz_*). Hence the total *MM* *d*-population is higher by one electron than in the case of DFT. Still, the experimental *MM* population is relevant to multipole moment decomposition and should lead to a Ti charge of one, while the *MM* QTAIM charge of Ti is two. In the case of DFT *d*-orbital natural populations, the sum of these *d*-populations is close to 2.0, although the Ti charge from DFT based natural population analysis is 1.24 e^−^. We have also optimized the local coordinate system for the titanium atom by minimizing the *MM* populations of *d*_*z*^2^_ and *d*_*x*^2^__−__*y*^2^_ orbitals (*d*_*z*^2^_ + *d*_*x*^2^__−__*y*^2^_) using the program *ERD* (Sabino & Coppens, 2002 ▸). The obtained *d*_*x*^2^__−__*y*^2^_ orbital population was 0.5113 e^−^ [the Ti—O(3), Ti—O(4), Ti—N(1) and Ti—N(2) coordination bonds], the *d_xy_* orbital population was 0.7351 e^−^ (non-bonding orbital) and *d*_*z*^2^_ orbital population was 0.7086 e^−^ (axial interaction with the peroxide anion).

### 3.3. Theoretical assessment of Ti–peroxo interactions   

First of all, we focused on the O_2_ moiety itself. One can compare the O—O QTAIM bond descriptors in the title compound with references such as O_2_, O_2_^−^ and O_2_^2−^, see Table S7 (O_2_^2−^ in the triplet spin state is unstable). We may conclude that the B3LYP and CCSD data are in a reasonable agreement. The bond length increases with the negative charge *q* of the O_2_^*q*^ system, and hence the BCP electron density (*i.e.* bond strength) decreases. This is in line with occupying the π*(O_2_) orbitals, with O_2_^2−^ having fully populated π and π* MOs. Actually, the O(1)—O(2) bond length in the complex studied is in between the O–O bond length of the free O_2_^−^ and O_2_^2−^ systems, compare Tables 2 ▸ and S7. Similar relations hold for BCP electron density and the Laplacian, although the O(1)—O(2) BCP Laplacian in the complex under study is closer to O_2_^2−^. Hence, upon Ti^IV^←O_2_^−^ coordination, the charge transfer from the π(O_2_) and π*(O_2_) orbitals leads to shortening of the O—O distance because of a lower repulsion between lower charge densities at particular oxygen atoms. Note also that the BCP Laplacian in ^1^O_2_^2−^ is positive, at both CCSD and B3LYP levels of theory.

**Figure 4:**
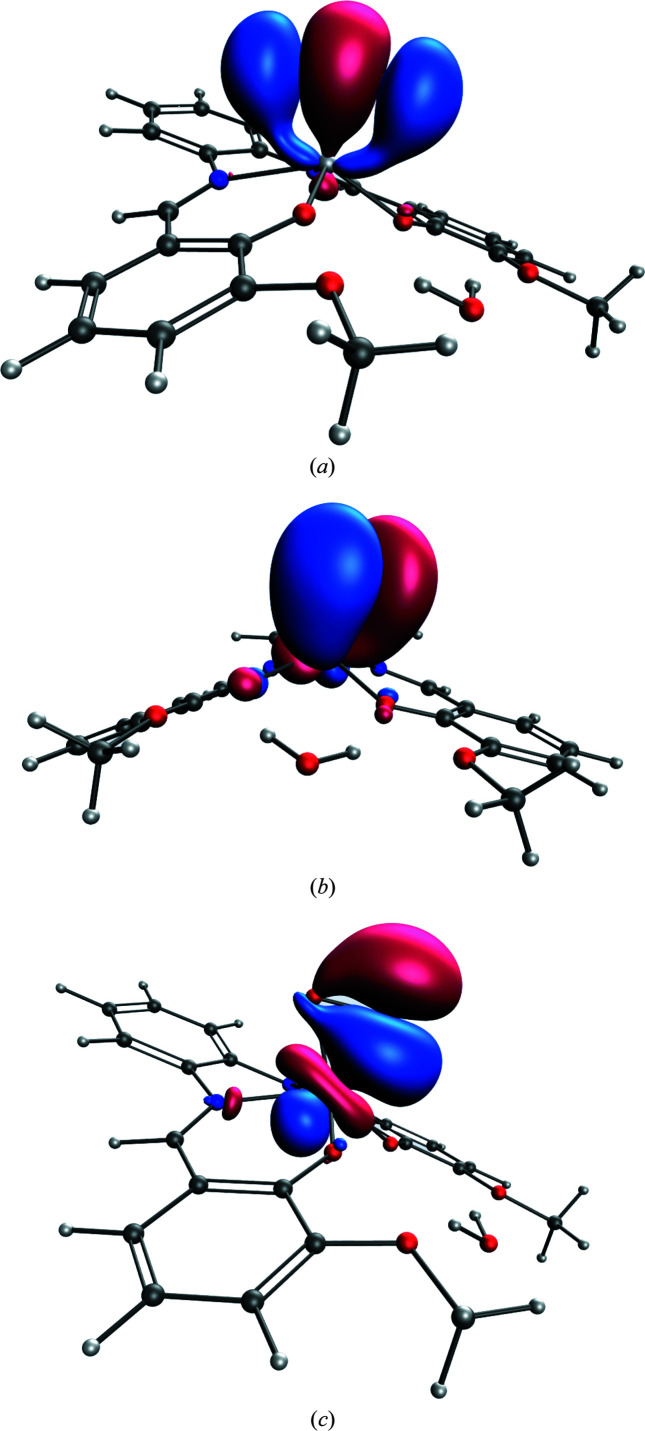
(*a*) σ O(1)—O(2)-like DAFH eigenvector (1.95); (*b*) *p_y_* O(1)-like DAFH eigenvector (1.82); (*c*) *p_z_* O(1)-like DAFH eigenvector (1.64) (DAFH eigenvalues are shown in parentheses).

To obtain further insight into the Ti^IV^←O_2_^2−^ coordination in the complex under study, DAFH analysis was performed, defining O_2_ as the domain to inspect the bonding interactions (DAFH eigenvectors) which are retained (eigenvalues close to 2) or split (eigenvalues <2, but >0.05) because of the domain choice itself. In the case of the O_2_ domain, one finds nine such DAFH eigenvectors. Four of the DAFH eigenvectors can be assigned to the 1*s*- and 2*s*-like densities on the oxygens (eigenvalues > 1.98), we will exclude these from consideration. Instead, we show the three distinguished contributions, *i.e.* the σ_O(1)—O(2)_ and the *p_z_* O(1) and the *p_y_* O(1)-like DAFH eigenvectors, see Fig. 4 ▸. DAFH *p_z_-* and *p_y_*-like eigenvectors of O(2) and O(1) are similar, hence those of O(2) are not shown in any detail. From the eigenvalues and pictorial representation of DAFH eigenvectors, we may conclude that the σ_O(1)—O(2)_ bond does not really contribute outside the peroxo-like moiety. The double-occupied *p_y_*(O) orbitals [perpendicular to the Ti—O(1)—O(2) plane] contribute less to the coordination bond than the *p_z_*(O) orbitals [in the Ti—O(1)—O(2) plane]. Apparently, DAFH analysis breaks the Ti^IV^←O_2_^2−^ interactions into individual Ti^IV^←O(1) and Ti^IV^←O(2) eigenvectors, which have a small π-like O(1)—O(2) contribution, but which are in different phases for the O(1) and O(2) atoms. Hence instead of the formal π_y,z_ and π*_y,z_ orbitals we obtained nonbonding *p_y_* and p_z_ orbitals in the DAFH representation which mediate the dative interactions (coordination bonds) between Ti and O(1)—O(2). The polarization of the σ_O(1)—O(2)_ DAFH eigenvector and the dative character of *p_z_*(O) DAFH eigenvectors [Figs. 4 ▸(*a*) and 4(*c*)] are in line with the shape of the *MM* deformation density and Laplacian maps being shifted or pointed from O(1)—O(2) towards Ti (Fig. 2 ▸).

## 4. Conclusions   

By means of the charge density study presented here we proved that, in the title compounds, the O—O bonding electron density is significantly shifted towards the central titanium atom. The BCP Laplacians of the O—O bonds in the O_2_ molecule, as well as in the title molecule are positive. This indicates that VSCC belongs to the area which is outside the O—O bond. The difference is that the VSCC in peroxo-complexes is asymmetric with respect to the O—O bond and symmetric for the O_2_ molecule. The O—O bond in the peroxide complex is weakened and therefore could be susceptible to a nucleophilic addition reaction. The differences between the experimental and theoretical electronic structure clearly show that, in the case of the stabilizing effect of molecules in the surroundings, this attenuating effect of O—O binding could be increased. Properties of the O—O moiety could be modified by suitable surrounding of the central titanium atom. In the case of different central atoms, the behavior of similar complexes could be comparable. Shifting the electron density is just a first step in the chain of subsequent reaction mechanisms. By modifying the supporting ligand, both electrophilic and nucleophilic reactions can take place. To the best of our knowledge, this is the first example of a charge density study of a coordination compound in which a peroxo anion is bonded to a 3*d* central atom. Interestingly, titanium has been found in a number of marine organisms which makes this metal important from a biological point of view. In fact, several titanium compounds have been shown to possess anticancer properties, enzyme inhibiting and antibacterial activities. Budo-titane and titanocene dichloride have been used in human anticancer clinical trials, however, to date have not reached clinical use. The main problem with the use of these compounds is the dose limit toxicity and solubility. Recently Obeid *et al.* (2012 ▸) reported Schiff-base titanium(IV) complexes with promising anticancer and antibacterial properties. In this work, authors proposed that DNA cleavage activity of Ti(IV) complexes was achieved *via* ROS-induced oxidative damage, predominantly by the DNA damaging activity of the hydroxyl radical. The Schiff base Ti(IV) peroxo complex studied in this work may exhibit similar biological (anticancer) properties, which may be enhanced by the presence of a peroxo group capable of participating in free-radical DNA damaging cascades. Related studies are underway. Differences between experimental and theoretical results, in which the properties of the isolated molecule and the molecule in the crystal are similar, are a good inspiration to improve the model of the molecular system. A theoretical study of the modified environment of the donor atoms can be used to tune the nature of the O—O bond. This electronic structure could be used as a possible model structure of the reaction center for hemoglobin or other metalloproteins.

## 5. Related literature   

The following reference is cited in the supporting information: Allen & Bruno (2010 ▸).

## Supplementary Material

Crystal structure: contains datablock(s) global, I, multipole. DOI: 10.1107/S205225252100004X/lq5035sup1.cif

Structure factors: contains datablock(s) I. DOI: 10.1107/S205225252100004X/lq5035sup2.hkl

Supporting information file. DOI: 10.1107/S205225252100004X/lq5035sup3.pdf

CCDC references: 2053715, 2062601

## Figures and Tables

**Table 1:** Experimental details

Crystal data	
Chemical formula	[Ti(C_22_H_18_N_2_O_6_)]·H_2_O
Formula weight, *Z*	472.30, 2
*F*(000)	488
Temperature (K)	100.0 (1)
Crystal size (mm)	0.145 × 0.192 × 0.476
*a* (Å)	7.4798 (2)
*b* (Å)	12.2525 (4)
*c* (Å)	12.6131 (4)
α (°)	115.637 (2)
β (°)	91.030 (3)
γ (°)	103.625 (3)
*V* (Å^3^)	1003.29 (6)
Space group	No. 2, *P* 1
Wavelength (Å)	0.56083
μ (mm^−1^)	0.254
Scan type	Ω scans
Crystal size (mm)	0.045 × 0.082 × 0.150
Max sin(θ/λ) (Å^−1^)	1.253
Range of indices for *h*	−18, +17=
Range of indices for *k*	±30
Range of indices for *l*	±31
No. of measured diffractions	80862
Redundancy (all)	10.08
*R*_int_ (for resolution 0.65 Å)	0.0696
*R*(σ) (for resolution 0.65 Å)	0.0307
Crystal to detector (mm)	40
X-ray tube (kV), μA	50, 880
	
Multipole refinement on *F* ^2^	
*R*(*F*), *wR*(*F*), GOOF	0.0385, 0.0172, 0.9098
*R*(*F* ^2^), *wR*(*F* ^2^), GOOF*w*	0.0486, 0.0329, 0.9122
*N*_ref_/*N*_v_	51.6539
Δρ_max_, Δρ_min_ (eÅ^−3^)	2.38, −0.82

**Table 2:** Selected AIM electron density properties at bond critical points (bond path length *d*_12_ = *d*_1_ + *d*_2_)

		Bond		BCP characteristics					
Geometry	Method	Atom 1	Atom 2	*d*_12_ (Å)	ρ_BCP_ (e Å^3^)	∇^2^ρ_BCP_ (e Å^5^)	ɛ	*d*_1_ (Å)	*d*_2_ (Å)
Exp.	Exp.	Ti	O(1)	1.8777	0.88 (2)	18.73 (4)	0.81	0.9553	0.9224
Exp.	Exp.†	Ti	O(1)	1.8795	0.89 (2)	19.04 (4)	1.03	0.9474	0.9320
Exp.	DFT	Ti	O(1)	1.8764	0.9161	11.6452	0.073	0.9519	0.9245
DFT	DFT	Ti	O(1)	1.8272	1.0378	12.8197	0.086	0.9296	0.8976
Exp.	Exp.	Ti	O(2)^*^	1.8885	0.87 (2)	17.64 (4)	0.82	0.9576	0.9308
Exp.	Exp.†	Ti	O(2)^*^	1.8922	0.82 (2)	17.00 (4)	1.05	0.9489	0.9434
Exp.	DFT	Ti	O(2)^*^	1.8884	0.8823	11.4024	0.052	0.9566	0.9318
DFT	DFT	Ti	O(2)^*^	1.8451	0.9835	12.8530	0.059	0.9372	0.9079
Exp.	Exp.	Ti	O(3)	1.8966	0.79 (1)	15.95 (4)	0.10	0.9545	0.9421
Exp.	Exp.†	Ti	O(3)	1.8989	0.82 (1)	13.92 (3)	0.13	0.9556	0.9433
Exp.	DFT	Ti	O(3)	1.9009	0.8122	13.6067	0.057	0.9565	0.9444
DFT	DFT	Ti	O(3)	1.9101	0.7439	12.3081	0.061	0.9599	0.9502
Exp.	Exp.	Ti	O(4)	1.9235	0.69 (1)	14.59 (3)	0.19	0.9739	0.9496
Exp.	Exp.†	Ti	O(4)	1.9262	0.71 (1)	13.63 (3)	0.20	0.9745	0.9517
Exp.	DFT	Ti	O(4)	1.9275	0.7689	12.9308	0.032	0.9737	0.9538
DFT	DFT	Ti	O(4)	1.9560	0.6732	11.1183	0.026	0.9871	0.9689
Exp.	Exp.	O(1)	O(2)	1.5091	2.45 (3)	16.16 (4)	0.06	0.7701	0.7390
Exp.	Exp.†	O(1)	O(2)	1.5118	2.32 (2)	11.93 (6)	0.05	0.7638	0.7479
Exp.	DFT	O(1)	O(2)	1.5052	1.5914	5.2606	0.070	0.7526	0.7523
DFT	DFT	O(1)	O(2)	1.4595	1.8050	3.9597	0.071	0.7303	0.7292

† Two different scattering factors for oxygen atoms.
