# Supplementary material for: Electronic structure of Schiff-base peroxo{2,2′-[1,2-phenyl­enebis(nitrilo­methanylyl­idene)]bis­(6-meth­oxy­phenolato)}titanium(IV) monohydrate: a possible model structure of the reaction center for the theoretical study of hemoglobin
Source: IUCrJ. 2021 Feb 18;8(Pt 2):295–304. doi: 10.1107/S205225252100004X (PMC7924236; doi:10.1107/S205225252100004X)
Supplement: Supplementary file 3 [file m-08-00295-sup3.pdf]

IUCrJ

Volume 8 (2021)

Supporting information for article:

Electronic structure of Schiff-base peroxo{2,2′-[1,2-phenylenebis(nitrilomethanylylidene)]bis(6-methoxyphenolato)}titanium(IV) monohydrate: a possible model structure of the reaction center for the theoretical study of hemoglobin

Júlia Adamko Kožíšková, Martin Breza, Marián Valko, Peter Herich, Lukáš Bučínsky and Jozef Kožíšek

S1 Multipole model refinement details.

At first, the scale factor was refined. Later, the atomic positions and the thermal parameters were refined with the bond lengths between carbon, or oxygen and hydrogen atoms kept fixed at average values determined by neutron diffraction studies, taken from the study of Allen *et al.* (Allen & Bruno, 2010). Bond lengths were of 1.077, 1.083 and 0.983 Å, for C—C(sp^3)—H_3; C(sp^2)—H (benzene); O—H (water), respectively. In the next step the κ refinement was performed, where only monopole populations along with a single κ value for all non-hydrogen atoms were refined (the κ value for hydrogens was set to 1.2). Subsequently, monopole populations were fixed and all other model parameters, as κ values, dipoles, quadrupoles, octupoles and for titanium atom also hexadecapoles, were progressively refined. After convergence achieving, all multipole parameters of all the atoms were refined simultaneously. Reflections up to $\sin\theta/\lambda \leq 1.253$ Å^{-1} have been taken into account.

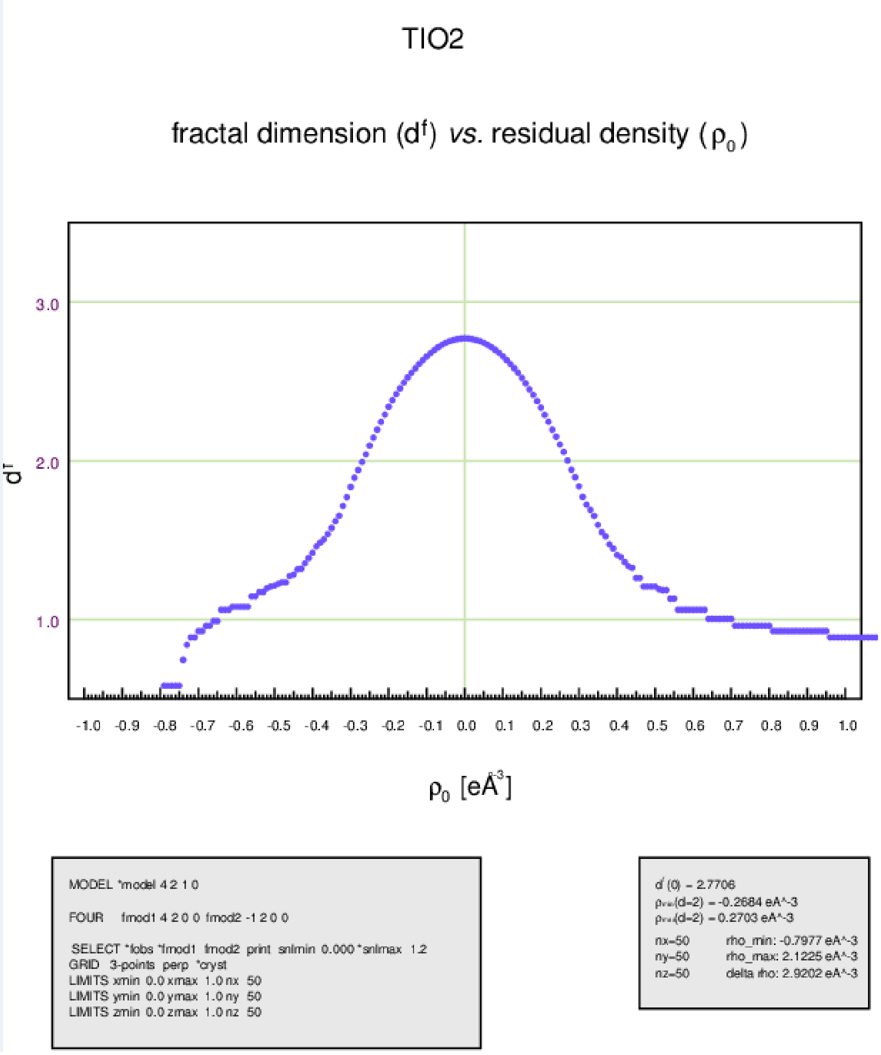

Extrema of residual densities were evaluated to judge the quality of the MM fit. To analyze the quality of the data and the MM refinement, the error analyses by the normal probability plot, variation of the scale factor and fractal dimension plot of the residual density were depicted (Fig. S1).

Figure S1. Fractal analysis of the residual density (Meindl & Henn, 2008).

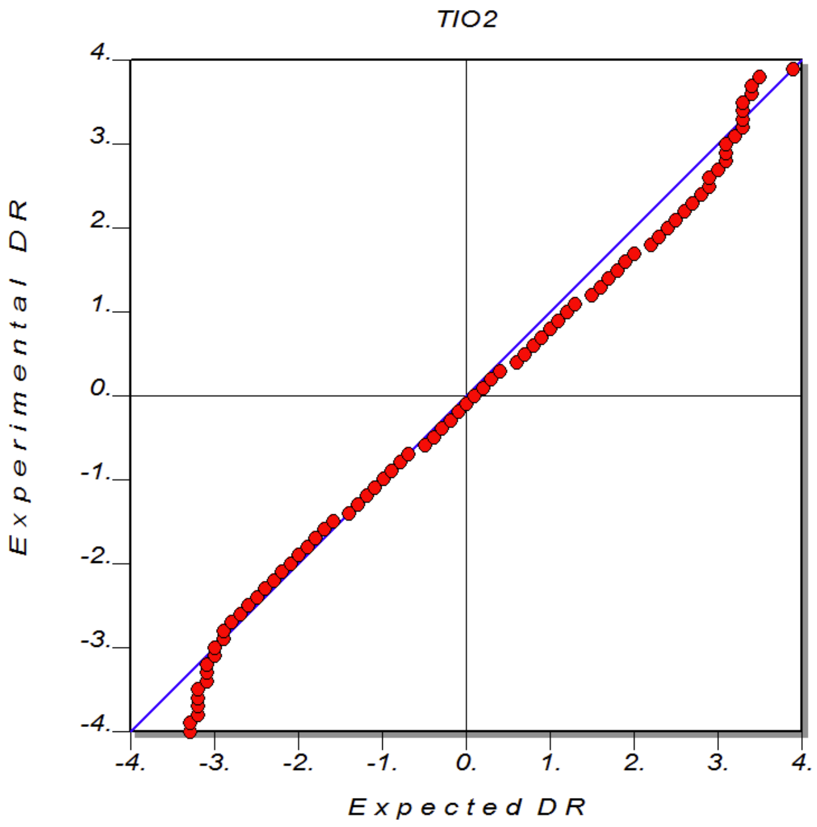

Figure S2. Normal probability distribution plot (Abrahams & Keve, 1971).

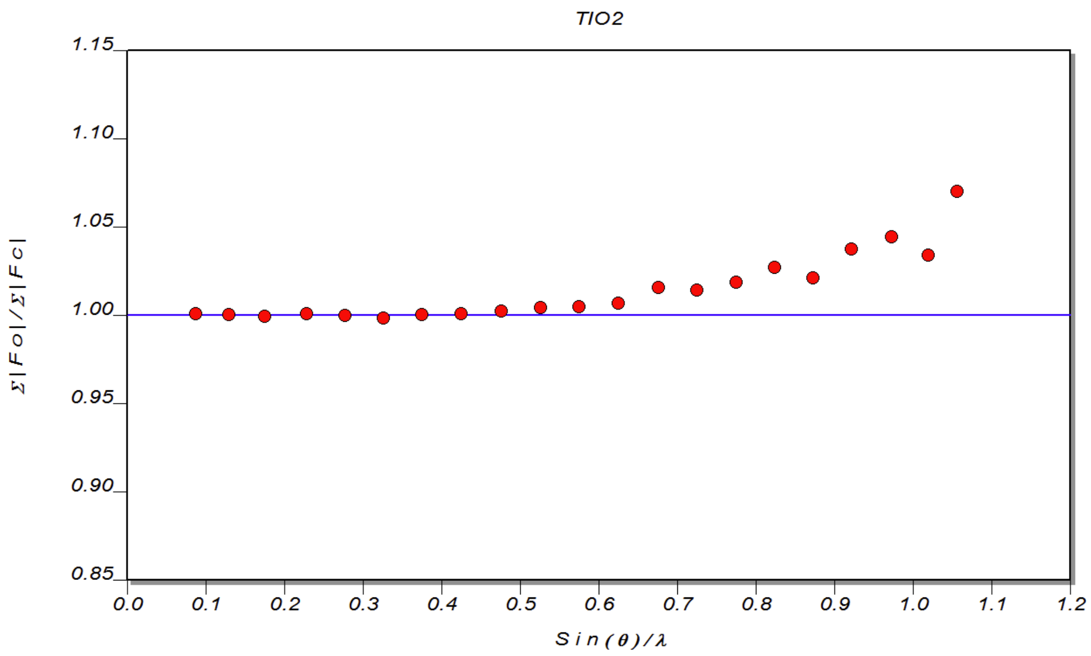

Figure S3. Variation of scale factors (Farrugia, 2012).

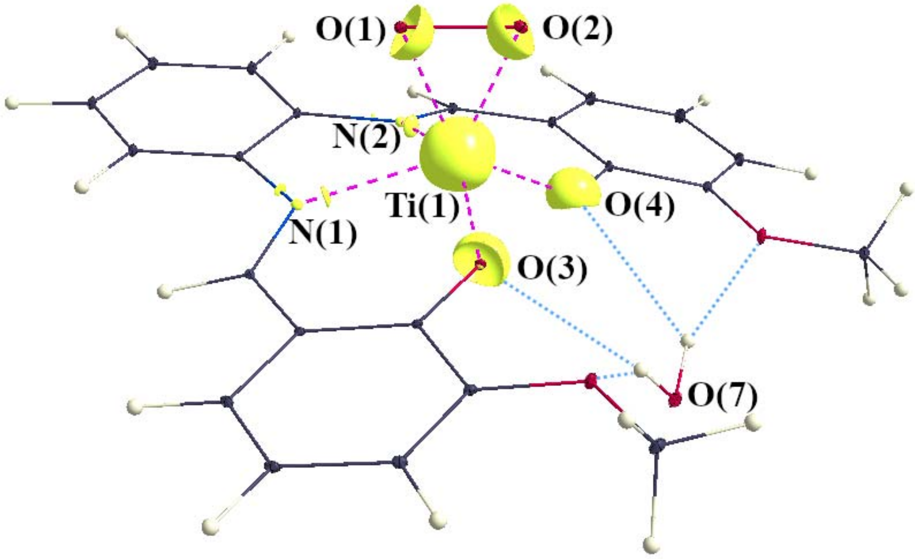

Figure S4. Three-dimensional plot (Hübschle & Dittrich, 2011) of the Laplacian of electron density around Cu at the isosurface value of 70 e Å-5.

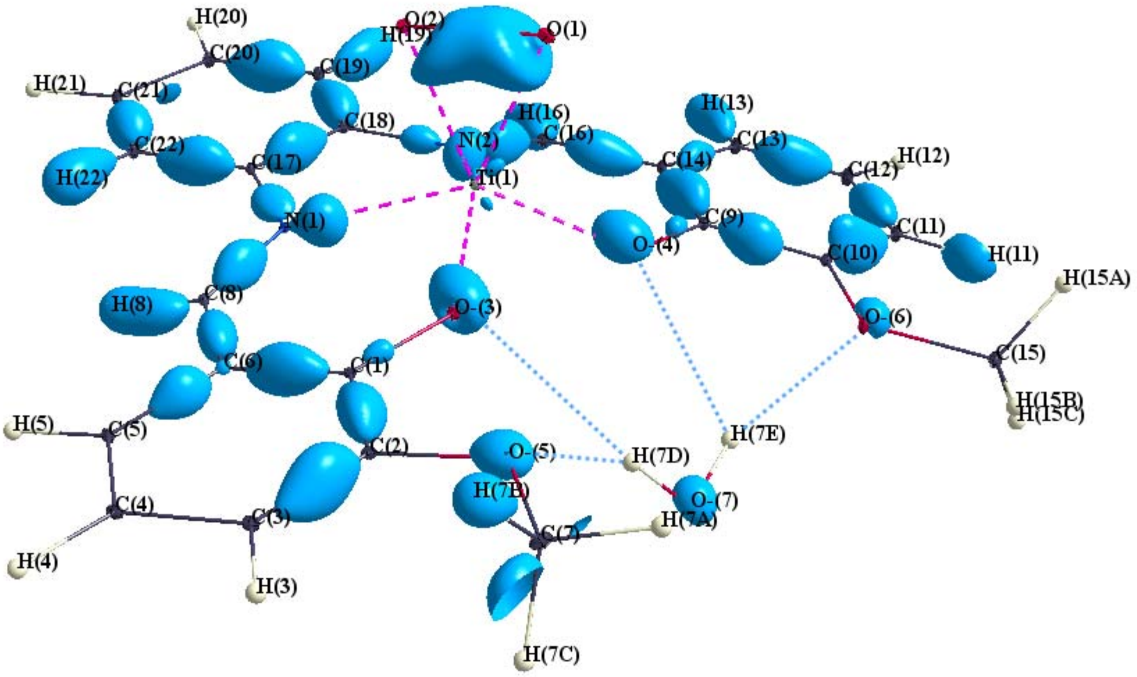

Figure S5. Three-dimensional plot (Hübschle & Dittrich, 2011) of the static electrondeformation density at the isosurface value of 0.4 e Å-3.

Table S1 Hydrogen bonds, distances [Å] , angles [º] and AIM properties

**contact (O···H−X)**	**Geometry**	**Method**	**R(O·····X) [Å]**	**R(O···H) [Å]**	**O···H−X [º]**	**ρ_{BCP} [e/Å^3]**	**$\nabla^2\rho_{BCP}$ [e/Å^5]**	**ε**
O(3)…H(7d)-O(7)	Exp.	Exp.	3.1131(15)	2.3553(16)	133.34(4)	0.069(2)	1.022(2)	0.14
	Exp.	DFT	dtto	dtto	dtto	0.0730	0.9846	0.304
	DFT	DFT	3.243	2.438	140.5	0.0616	0.8480	0.685
O(4)…H(7e)-O(7)	Exp.	Exp.	3.1894(18)	2.5496(18)	128.38(4)	0.061(1)	0.817(1)	0.33
	Exp.	DFT	dtto	dtto	dtto	0.0580	0.7803	0.579
	DFT	DFT	3.251	2.436	141.8	0.0620	0.8575	0.704
O(5)…H(7d)-O(7)	Exp.	Exp.	3.0725(17)	2.1731(17)	151.43(5)	0.079(4)	1.472(2)	0.10
	Exp.	DFT	dtto	dtto	dtto	0.0981	1.3902	0.056
	DFT	DFT	2.884	2.030	146.2	0.1405	2.0408	0.059
O(6)…H(7e)-O(7)	Exp.	Exp.	3.0402(18)	2.0938(19)	161.03(5)	0.108(6)	1.664(4)	0.02
	Exp.	DFT	dtto	dtto	dtto	0.1174	1.6729	0.044
	DFT	DFT	2.869	2.011	146.7	0.1432	2.1346	0.050
O(1)…H(4)$^{\$}$-C(4)$^{\$}$	Exp.	Exp.	3.4451(14)	2.7729(15)	134.87(4)	0.044(1)	0.529(0)	0.45
O2…H(3)$^{\$}$-C(3)$^{\$}$	Exp.	Exp.	3.1573(13)	2.3778(13)	129.37(4)	0.076(2)	0.975(1)	0.11
O1…H(15B)$^{\$\$}$-C(15)$^{\$\$}$	Exp.	Exp.	3.0459(16)	2.6635(16)	109.96(4)	0.057(1)	0.592(1)	0.17
O1…H(19)$^{\$\$\$}$-C(19)$^{\$\$\$}$	Exp.	Exp.	3.1501(14)	2.2623(15)	164.66(4)	0.104(7)	1.565(3)	0.05
O2…H(13)$^{\$\$\$}$-C(13)$^{\$\$\$}$	Exp.	Exp.	3.3527(16)	2.5437(16)	137.98(3)	0.053(3)	0.755(1)	0.03
O2… H(16)$^{\$\$\$}$-C(16)$^{\$\$\$}$	Exp.	Exp.	3.4152(14)	2.7158(14)	112.24(2)	0.043(1)	0.497(0)	0.94
O(7)…H(20)*-C(20)*	Exp.	Exp.	3.3293(17)	2.4916(18)	134.45(4)	0.050(2)	0.720(1)	0.08
			-	-	-	-	-	-
O(7)…H(8)**-C(8)**	Exp.	Exp.	3.4840(17)	2.5149(18)	156.97(4)	0.063(4)	0.779(1)	0.16
			-	-	-	-	-	-

Symmetry code used: $^{\$}$) 2-X, 1-Y, 1-Z; $^{\$\$}$) 1-X, -Y, -Z; $^{\$\$\$}$) 1-X, 1-Y, -Z; ^{i}) X, -1+Y, Z; ^{ii}) 1-X, 1-Y, 1-

Table S2 Setected interatomic distances [Å], angles [º].

	**MM**	DFT		**MM**	DFT
Ti-O(1)	1.8709(12)	1.820	Ti-O(1)-O(2)	66.78(7)	67.1

Ti-O(2)	1.8827(11)	1.837	Ti-O(2)-O(1)	65.95(6)	65.9
Ti-O(3)	1.8962(11)	1.906	O(1)-Ti-O(2)	47.27(5)	47.0
Ti-O(4)	1.9249(10)	1.952	O(3)-Ti-O(4)	92.56(5)	94.1
Ti-N(1)	2.1601(10)	2.205	O(3)-Ti-N(1)	84.53(4)	82.6
Ti-N(2)	2.1567(10)	2.182	O(3)-Ti-N(2)	145.78(4)	142.8
O(1)-O(2)	1.5050(16)	1.459	O(4)-Ti-N(1)	134.04(4)	130.2
			O(4)-Ti-N(2)	82.83(4)	81.5
			N(1)-Ti-N(2)	75.04(4)	72.9

Table S3 AIM electron density properties at bond critical points (bond path length $d_{12} = d_1 + d_2$)

Geometry	Method	Bond		BCP characteristics					
		Atom 1	Atom 2	d_{12} [Å]	ρ_{BCP} [e/Å^3]	$\nabla^2\rho_{BCP}$ [e/Å^5]	ε	d_1 [Å]	d_2 [Å]
Exp.	Exp.	Ti	O(1)	1.8777	0.88(2)	18.73(4)	0.81	0.9553	0.9224
Exp.	Exp.#	Ti	O(1)	1.8795	0.89(2)	19.04(4)	1.03	0.9474	0.9320
Exp.	DFT	Ti	O(1)	1.8764	0.9161	11.6452	0.073	0.9519	0.9245
DFT	DFT	Ti	O(1)	1.8272	1.0378	12.8197	0.086	0.9296	0.8976
Exp.	Exp.	Ti	O(2)*	1.8885	0.87(2)	17.64(4)	0.82	0.9576	0.9308
Exp.	Exp.#	Ti	O(2)*	1.8922	0.82(2)	17.00(4)	1.05	0.9489	0.9434
Exp.	DFT	Ti	O(2)*	1.8884	0.8823	11.4024	0.052	0.9566	0.9318
DFT	DFT	Ti	O(2)*	1.8451	0.9835	12.8530	0.059	0.9372	0.9079
Exp.	Exp.	Ti	O(3)	1.8966	0.79(1)	15.95 (4)	0.10	0.9545	0.9421
Exp.	Exp.#	Ti	O(3)	1.8989	0.82(1)	13.92 (3)	0.13	0.9556	0.9433
Exp.	DFT	Ti	O(3)	1.9009	0.8122	13.6067	0.057	0.9565	0.9444
DFT	DFT	Ti	O(3)	1.9101	0.7439	12.3081	0.061	0.9599	0.9502
Exp.	Exp.	Ti	O(4)	1.9235	0.69(1)	14.59 (3)	0.19	0.9739	0.9496
Exp.	Exp.#	Ti	O(4)	1.9262	0.71(1)	13.63 (3)	0.20	0.9745	0.9517
Exp.	DFT	Ti	O(4)	1.9275	0.7689	12.9308	0.032	0.9737	0.9538
DFT	DFT	Ti	O(4)	1.9560	0.6732	11.1183	0.026	0.9871	0.9689
Exp.	Exp.	Ti	N(1)	2.1626	0.45(1)	7.42(2)	0.09	1.0648	1.0978
Exp.	Exp.#	Ti	N(1)	2.1610	0.45(1)	7.29(2)	0.11	1.0653	1.0957
Exp.	DFT	Ti	N(1)	2.1629	0.4806	5.6860	0.095	1.0568	1.1047
DFT	DFT	Ti	N(1)	2.2077	0.4332	5.1658	0.065	1.0767	1.1310
Exp.	Exp.	Ti	N(2)	2.1570	0.47(1)	7.20(2)	0.14	1.0695	1.0875
Exp.	Exp.#	Ti	N(2)	2.1571	0.47(1)	7.15(2)	0.16	1.0694	1.0878
Exp.	DFT	Ti	N(2)	2.1572	0.4865	5.5724	0.068	1.0517	1.1055
DFT	DFT	Ti	N(2)	2.1831	0.4574	5.2105	0.053	1.0615	1.1216
Exp.	Exp.	O(1)	O(2)	1.5091	2.45(3)	16.16(4)	0.06	0.7701	0.7390
Exp.	Exp.#	O(1)	O(2)	1.5118	2.32(2)	11.93(6)	0.05	0.7638	0.7479
Exp.	DFT	O(1)	O(2)	1.5052	1.5914	5.2606	0.070	0.7526	0.7523
DFT	DFT	O(1)	O(2)	1.4595	1.8050	3.9597	0.071	0.7303	0.7292
Exp.	Exp.	O(3)	C(1)	1.3176	2.22(5)	-23.92(3)	0.05	0.8374	0.4802
Exp.	Exp.#	O(3)	C(1)	1.3175	2.38(3)	-20.8(1)	0.05	0.7388	0.5786
Exp.	DFT	O(3)	C(1)	1.3190	2.1199	-8.4594	0.007	0.8683	0.4507
DFT	DFT	O(3)	C(1)	1.3102	2.1491	-7.0934	0.006	0.8642	0.4459
Exp.	Exp.	O(4)	C(9)	1.3177	2.20(5)	-25.73(3)	0.20	0.8443	0.4734
Exp.	Exp.#	O(4)	C(9)	1.3152	2.38(3)	-22.0(1)	0.16	0.7451	0.5700
Exp.	DFT	O(4)	C(9)	1.3165	2.1458	-9.2366	0.012	0.8655	0.4510
DFT	DFT	O(4)	C(9)	1.3018	2.2070	-7.6743	0.008	0.8579	0.4438
Exp.	Exp.	N(1)	C(8)	1.3007	2.59(4)	-27.62(2)	0.23	0.7367	0.5639
Exp.	Exp.#	N(1)	C(8)	1.3017	2.54(4)	-27.3(2)	0.21	0.7533	0.5484
Exp.	DFT	N(1)	C(8)	1.3041	2.3996	-20.8494	0.135	0.8340	0.4701
DFT	DFT	N(1)	C(8)	1.3000	2.4161	-20.7356	0.137	0.8321	0.4679
Exp.	Exp.	N(1)	C(17)	1.4192	1.93(4)	-16.45(2)	0.15	0.8569	0.5623

Exp.	Exp.#	N(1)	C(17)	1.4194	1.95(4)	-16.5(1)	0.16	0.8464	0.5731
Exp.	DFT	N(1)	C(17)	1.4194	1.9282	-19.2565	0.065	0.8613	0.5581
DFT	DFT	N(1)	C(17)	1.4125	1.9526	-19.6905	0.060	0.8626	0.5499
Exp.	Exp.	N(2)	C(16)	1.3057	2.56(4)	-30.72(2)	0.34	0.7600	0.5457
Exp.	Exp.#	N(2)	C(16)	1.3066	2.52(4)	-29.7(2)	0.34	0.7641	0.5424
Exp.	DFT	N(2)	C(16)	1.3071	2.3872	-20.9700	0.125	0.8355	0.4716
DFT	DFT	N(2)	C(16)	1.3043	2.3956	-20.8987	0.128	0.8340	0.4703
Exp.	Exp.	N(2)	C(18)	1.4182	1.90(4)	-14.57(1)	0.05	0.8438	0.5744
Exp.	Exp.#	N(2)	C(18)	1.4183	1.92(4)	-14.4(1)	0.06	0.8284	0.5899
Exp.	DFT	N(2)	C(18)	1.4173	1.9377	-19.4469	0.065	0.8608	0.5564
DFT	DFT	N(2)	C(18)	1.4147	1.9455	-19.5966	0.060	0.8620	0.5527

Exp# two different scattering factors for O atoms

Table S4 AIM atomic charges and atomic volumes

Geometry	Method	Ti	O(1)	O(2)	O(3)	O(4)	O(5)	O(6)	N(1)	N(2)	O(7)	C(1)	C(9)	C(17)	C(18)
								charge [e⁻]							
Exp.	Exp.	2.05	-0.21	-0.07	-1.23	-1.18	-1.25	-1. 06	-1.02	-1.05	-1.46	0.53	0.60	0.21	0.33
Exp.	Exp.#	2.07	-0.27	-0.12	-1.13	-1.10	-1.17	-0.99	-1.01	-1.02	-1.27	0.40	0.40	0.20	0.28
Exp.	DFT	2.14	-0.50	-0.52	-1.13	-1.13	-1.04	-1.03	-1.18	-1.19	-1.09	0.68	0.69	0.35	0.36
DFT	DFT	2.14	-0.47	-0.49	-1.14	-1.15	-1.05	-1.05	-1.19	-1.19	-1.12	0.69	0.71	0.36	0.36
								V001 [Å^3]							
Exp.	Exp.	9.47	18.1	17.0	16.8	16.9	14.8	14.4	13.4	13.9	25.0	9.1	8.8	9.9	9.8
Exp.	Exp.#	9.16	18.2	17.1	17.9	18.1	16.3	16.0	13.2	13.6	26.7	9.5	9.3	9.9	9.9
Exp.	DFT	8.5	21.8	20.59	17.0	17.1	15.8	15.7	14.5	14.8	28.6	9.5	9.4	10.3	10.3
DFT	DFT	8.4	17.3	16.7	15.7	15.6	13.5	13.5	13.4	13.5	28.3	9.5	9.4	10.1	10.1

Exp# two different scattering factors for O atoms

Table S5 Non-covalent interactions, distances [Å] and AIM properties

**contact, (X···Y)**		**R(X···Y), [Å]**	**ρ_{BCP}, [e/Å^3]**	**$\nabla^2\rho_{BCP}$, [e/Å^5]**	ε	d_1 [Å]	d_2 [Å]
N(2)…C(4)^{ii}	Exp.	3.2385	0.043(1)	0.457(1)	0.84	1.6584	1.5801
C(1)…C(3)^{iii}	Exp.	3.3054	0.039(1)	0.407(1)	0.35	1.6987	1.6067
C(1)…C(8)^{ii}	Exp.	3.1260	0.047(1)	0.519(1)	7.33	1.6552	1.4708
C(2)…C(8)^{ii}	Exp.	3.5959	0.047(1)	0.519(1)	7.33	2.1251	1.4709
C(3)…C(17)^{ii}	Exp.	3.3510	0.036(1)	0.376(1)	0.72	1.7126	1.6384
C(6)…C(6)^{ii}	Exp.	3.3351	0.044(1)	0.460(1)	2.88	1.7510	1.5841

Symmetry code used: ^{ii}) 1- X, 1- Y, 1- Z; ^{iii}) 2- X, 1- Y, 1- Z

Table S6 Population of the *d*-orbitals on the central Ti atom [e^-]

Geometry	Method	$d_{x^2-y^2}$	d_{z^2}	d_{yz}	d_{xz}	d_{xy}	Σ
Exp.	Exp.	0.66(3)	0.72 (3)	0.65 (3)	0.53 (3)	0.55(3)	3.10
Exp.	ERD*	0.4893	0.7186	0.4799	0.6966	0.7128	3.0972
Exp.	Exp.$^{\#}$	0.69(3)	0.71(3)	0.64(3)	0.53(3)	0.56(3)	3.12
Exp.	ERD$^{\#}$*	0.5113	0.7086	0.4926	0.6774	0.7351	3.1250
Exp.	DFT	0.363	0.366	0.501	0.412	0.430	2.074
DFT	DFT	0.401	0.352	0.495	0.429	0.406	2.089

Exp$^{\#}$ two different scattering factors for O atoms; Exp* Sabino & Coppens, 2002

Table S7 DFT and CCSD QTAIM O-O bond descriptors in different O_2^q species

Method Species	B3LYP				CCSD			
	d_{O-O} [Å]	ρ_{BCP} [e/Å^3]	$\nabla^2\rho_{BCP}$ [e/Å^5]	ε_{BCP}	d_{O-O} [Å]	ρ_{BCP} [e/Å^3]	$\nabla^2\rho_{BCP}$ [e/Å^5]	ε_{BCP}
$^1O_2$	1.206	3.640	-18.302	0.000	1.209	3.621	-18.901	0.000
$^3O_2$	1.205	3.642	-18.467	0.000	1.201	3.693	-19.913	0.000
$^2O_2^-$	1.346	2.446	-3.166	0.005	1.339	2.498	-3.894	0.005
$^1O_2^{2-}$	1.569	1.302	5.394	0.000	1.570	1.296	6.125	0.000

References

Abrahams, S. C. & Keve, E. T. (1971). Acta Cryst. A27, 157–165.

Allen, F. H. & Bruno, I. J. (2010). Acta Cryst. B66, 380–386.

Farrugia, L. J. (2012). J. Appl. Cryst. 45, 849–854

Meindl, K. & Henn, J. (2008). Acta Cryst. A64, 404–418.

Sheldrick, G. M. (2015a). Acta Cryst. A71, 3–8.

Sheldrick, G. M. (2015b). Acta Cryst. C71, 3–8.

Volkov, A., Macchi, P., Farrugia, L. J., Gatti, C., Mallinson, P., Richter, T. & Koritsanszky, T. (2016). XD2016. University at Buffalo, State University of New York, Buffalo, NY, USA, http://www.chem.- gla.ac.uk/~louis/xd-home.
